# Two squamous cell carcinomas of the skin in a patient with Lynch syndrome

**DOI:** 10.1016/j.ijscr.2022.107822

**Published:** 2022-12-01

**Authors:** Oshan Basnayake, Umesh Jayarajah, Duminda Subasinghe, Kavinda Rajapakse, Thushan Beneragama, Sivasuriya Sivaganesh

**Affiliations:** a University Surgical Unit, Faculty of Medicine, University of Colombo, Sri Lanka; b Department of Surgery, Faculty of Medicine, University of Colombo, Sri Lanka; c Plastic and Reconstructive Surgical Unit, National Hospital of Sri Lanka, Colombo, Sri Lanka

**Keywords:** Lynch syndrome, Squamous cell carcinoma, Case report

## Abstract

**Introduction and importance:**

Although synchronous and metachronous tumours of the bowel are well known associations of Lynch syndrome, the association of skin malignancies in such patients are extremely rare.

**Case presentation:**

A 40-year-old Sri Lankan man with a strong family history of colorectal cancer had an extended right hemicolectomy for a moderately differentiated adenocarcinoma. Two months after surgery, he developed two discrete ulcerative skin lesions in the chin and occipital region which excision biopsy confirmed to be squamous cell carcinoma. After more than two years of follow-up, patient remains disease free.

**Clinical discussion:**

The Muir Torre variant of Lynch syndrome is characteristically associated with sebaceous adenomas and carcinomas, though occurence of squamous cell carcinomas are rare. In reported cases, defective mismatch repair genes associated with Lynch syndrome may suggest an increased predisposition for squamous cell carcinomas.

**Conclusion:**

Patients with Lynch syndrome should be educated on the importance of seeking an early medical consult for new skin lesions and raising awareness of this rare phenomenon for physicians involved in follow up is important.

## Highlights

• Patients with Lynch syndrome should be educated on the importance of seeking an early medical consult for new skin lesions
• Raising awareness of this rare phenomenon of associated skin cancer for physicians involved in follow up is needed
• The role of defective mismatch repair genes in predisposition should be analyzed further

## 1 Introduction

Inherited colorectal cancer (CRC) is common in patients who present young [1]. Among inherited cancer syndromes, Lynch syndrome is the commonest with a prevalence of 3 % in newly diagnosed patients with CRC [1]. Lynch syndrome occurs as a result of mutations in the DNA mismatch repair genes with an autosomal dominant inheritance pattern. *MSH2, MLH1,* MSH6, and PMS2 genes are found to be associated with Lynch syndrome. Although synchronous and metachronous large bowel tumours are well described, the association with skin malignancies is rare. We describe a patient with Lynch syndrome presenting with two malignant squamous cell lesions of the face and occiput. The work has been reported in line with the SCARE 2020 criteria [2].

## 2 Case presentation

A 40-year-old otherwise healthy Sri Lankan man presented with abdominal pain and reduced stool frequency of one month's duration. He gave a strong family history of bowel cancer. Two first degree and 6 second degree relatives had colonic cancer. His father and elder brother were diagnosed with colon cancer at the age of 54 and 42 years respectively. His father had 10 siblings and of them, 5 had colorectal cancers. The patient's grandfather was also diagnosed with colon cancer. His medical, drug and psychosocial history were unremarkable.

Colonoscopy and biopsy confirmed a moderately differentiated adenocarcinoma of the proximal transverse colon. Contrast enhanced computed tomography (CT) scan showed a mass in the proximal transverse colon without any evidence of locoregional spread or distant metastasis and the carcinoembryonic antigen (CEA) level was 2.6 ng/ml. After counseling, he opted for an extended right hemicolectomy with colonoscopic surveillance than a total colectomy. The final histology confirmed a moderately differentiated adenocarcinoma without any lymphovascular or perineural invasion and no metastasis to lymphnodes (T2N0Mx). Incidentally, multiple, caeseating epithelioid-type granulomas were found in the sub mucosa and mesenteric lymph nodes. He did not have clinical, biochemical (normal ESR, mantoux and interferon gamma) or radiological evidence (Chest X-ray and CT scan) of tuberculosis. He did not receive adjuvant chemotherapy but was commenced on a 9-month course of anti-tuberculosis treatment postoperatively with an intensive phase of 2 months (Rifampin, Isoniazid, Pyrazinamide, and Ethambutol) and continuation phase of 7 months (Rifampin and Isoniazid).

**Fig. 1:**
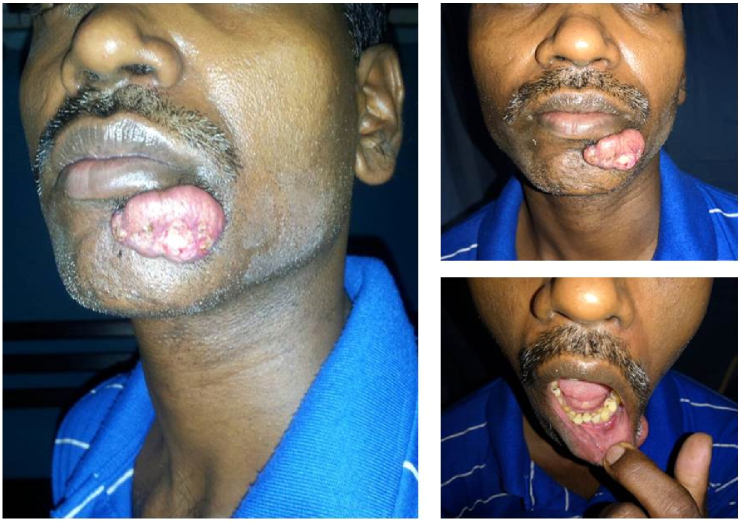
Pre-operative image of the squamous cell carcinoma near left lower lip.

**Fig. 2:**
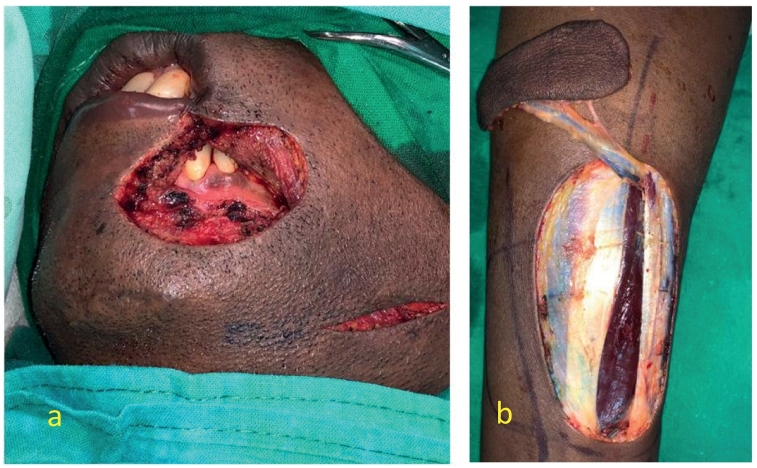
Intraoperative images; a- after excision of the lesion; b- harvested radial forearm flap.

**Fig. 3:**
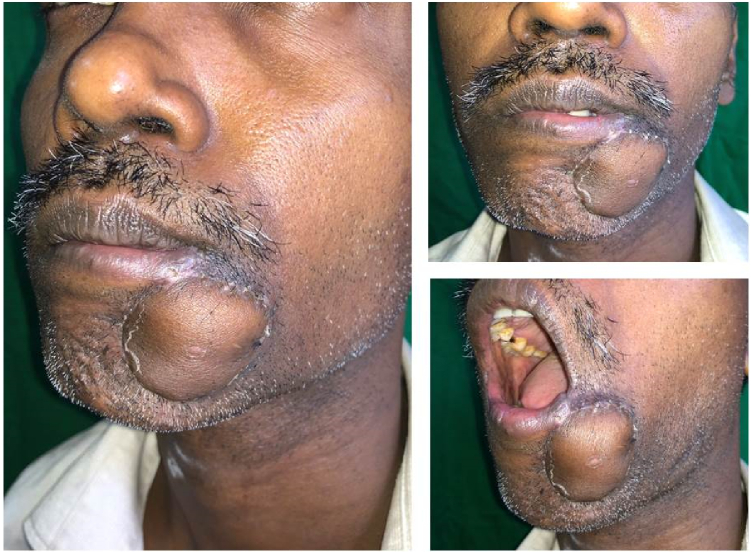
Post-operative images of reconstructed left chin region.

Two months following the hemicolectomy, he developed a rapidly enlarging lump over the chin (Fig. 1). This was mistakenly identified as a sebaceous cyst and excised in another unit. Biopsy revealed a well differentiated squamous cell carcinoma with an involved deep margin. There was no clinical or ultrasound evidence of cervical lymphadenopathy. A wide local excision of the tumor bed with a radial forearm free flap reconstruction was done (Figs. 2 and 3) without sentinel lymph node dissection. A month later he developed a similar nodular lesion in the occipital region which was excised and proved also to be a well differentiated squamous cell carcinomas with tumor free resection margins.

He was followed up with six monthly CEA, annual colonoscopy, annual CT scans and was disease free for more than 2 years after surgery.

## 3 Discussion

Lynch syndrome associated bowel cancers have right colonic predominance and an increased risk of metachronous and synchronous tumours compared with the sporadic type [3], [4]. The Amsterdam 1 criteria are used to identify patients with Lynch syndrome without genetic testing [5] while the less stringent Amsterdam 2 criteria were later introduced for research and clinical purposes. The revised Bethesda criteria is used to identify individuals who need genetic testing for microsatellite instability with a sensitivity and specificity of 82 % and 77 % respectively [5]. Our patient fulfilled the clinical criteria for the diagnosis of Lynch syndrome. Genetic analysis was not done due to its non-availability in the resource limited public health sector and high costs involved in sourcing it externally [6].

Lynch syndrome predisposes to extra-colonic tumours such as endometrial, renal pelvic, ovarian and brain tumours [7]. The Muir Torre variant of Lynch syndrome is characteristically associated with sebaceous adenomas, carcinomas, and keratoacanthomas [8]. However, the association with skin malignancies is rare with only four reported cases associated with squamous cell carcinomas of the skin [9], [10], [11], [12] (Table 1).

**Table 1:** Reported cases of squamous cell carcinomas of the skin in patients with Lynch syndrome.

	Publication year	Author	Age	Region	Genetic testing
1	2002	M Mathiak et al. [9]	N/A	N/A	Loss of MSH2
2	2015	Sorscher et al. [10]	54y	Anterior abdominal wall	Loss of expression of MLH-1 and PMS-2.
3	2017	C Kientz et al. [11]	41y	Nose	Partial loss of the expression of MSH2 and MSH6
4	2019	F Adan et al. [12]	33y	Face-cheek	Absent MSH2 and MSH6

Two reported cases had classical Lynch syndrome [10], [12] whereas the other two had the Muir Torre variant. Genetic analysis of the[9], [11] squamous cell carcinomas of the reported cases showed defective DNA mismatch repair genes frequently associated with Lynch syndrome (Table 1). Microsatellite instability is a characteristic feature of Lynch syndrome-associated cancers. However, the prevalence of microsatellite instability in squamous cell carcinoma was found to be very uncommon with a strong expression of MSH2 [13]. Unlike our patient, previous cases did not report on two synchronous squamous cell cancers occurring at anatomically distant sites. In general, synchronous lesions are those diagnosed within 6 months and metachronous lesions are those diagnosed after 6 months of the primary tumour [3]. Another case study reported a duodenal squamous cell carcinoma with loss of MSH2 and MSH6 gene expression associated with Lynch syndrome [14]. Defective mismatch repair genes associated with Lynch syndrome may suggest an increased predisposition for squamous cell carcinomas.

## 4 Conclusion

We report a rare occurrence of two synchronous squamous cell skin cancers within months of the diagnosis of Lynch syndrome. We recommend that patients with Lynch syndrome be educated on the importance of seeking an early medical consult for new skin lesions. We also wish to alert physicians managing these patients of this rare phenomenon.

## Abbreviations

CRC: colorectal cancer
CEA: carcinoembryonic antigen

## Availability of data and material

All data generated or analyzed during this study are included in this published article.

## Sources of funding

The authors received no financial support for the research, authorship, and/or publication of this article.

## Ethics approval

Not applicable.

## Consent

Written informed consent was obtained from the patient for publication of this case report and accompanying images. A copy of the written consent is available for review by the Editor-in-Chief of this journal on request.

## Author contribution

Author OB, UJ, DS, KR and TB contributed to collection of information and writing of the manuscript. Author SS contributed to writing and final approval of the manuscript. All authors read and approved the final version of the manuscript.

## Research registration

Not applicable.

## Guarantor

Sivasuriya Sivaganesh.

## Declaration of competing interest

The authors declare that they have no conflicts of interest.

## References

[1] Moreira L., Balaguer F., Lindor N., De la Chapelle A., Hampel H., Aaltonen L.A. (2012). Identification of lynch syndrome among patients with colorectal cancer. JAMA.

[2] Agha R.A., Franchi T., Sohrabi C., Mathew G., Kerwan A., Thoma A. (2020). The SCARE 2020 guideline: updating consensus surgical CAse REport (SCARE) guidelines. Int. J. Surg..

[3] Win A.K., Parry S., Parry B., Kalady M.F., Macrae F.A., Ahnen D.J. (2013). Risk of metachronous colon cancer following surgery for rectal cancer in mismatch repair gene mutation carriers. Ann. Surg. Oncol..

[4] Win A.K., Buchanan D.D., Rosty C., MacInnis R.J., Dowty J.G., Dite G.S. (2015). Role of tumour molecular and pathology features to estimate colorectal cancer risk for first-degree relatives. Gut.

[5] Giardiello F.M., Allen J.I., Axilbund J.E., Boland C.R., Burke C.A., Burt R.W. (2014). Guidelines on genetic evaluation and management of lynch syndrome: a consensus statement by the US multi-society task force on colorectal cancer. Gastroenterology.

[6] Jayarajah U., Abeygunasekera A.M. (2021). Cancer services in Sri Lanka: current status and future directions. J. Egypt. Natl. Canc. Inst..

[7] Møller P., Seppälä T., Bernstein I., Holinski-Feder E., Sala P., Evans D.G. (2017). Cancer incidence and survival in lynch syndrome patients receiving colonoscopic and gynaecological surveillance: first report from the prospective lynch syndrome database. Gut.

[8] John A.M., Schwartz R.A. (2016). Muir-torre syndrome (MTS): an update and approach to diagnosis and management. J. Am. Acad. Dermatol..

[9] Mathiak M., Rutten A., Mangold E., Fischer H.P., Ruzicka T., Friedl W. (2002). Loss of DNA mismatch repair proteins in skin tumors from patients with muir-torre syndrome and MSH2 or MLH1 germline mutations: establishment of immunohistochemical analysis as a screening test. Am. J. Surg. Pathol..

[10] Sorscher S. (2015). A case of squamous cell carcinoma of the skin due to the molecularly confirmed lynch syndrome. Hereditary Cancer Clin. Pract..

[11] Kientz C., Joly M.-O., Faivre L., Clemenson A., Dalac S., Lepage C. (2017). A case report of muir-torre syndrome in a woman with breast cancer and MSI-low skin squamous cell carcinoma. Hereditary Cancer Clin. Pract..

[12] Adan F., Crijns M., Dekker E., Bastiaansen B., Lapid O., Snaebjornsson P. (2019). A squamous cell carcinoma in a young woman with lynch syndrome. Familial Cancer.

[13] Gray S.E., Kay E.W., Leader M., Mabruk M.J. (2006). Enhanced detection of microsatellite instability and mismatch repair gene expression in cutaneous squamous cell carcinomas. Mol. Diagn. Ther..

[14] Amjad A.I., Singhi A.D., Balaban E.P., Dudley B., Brand R.E., Bahary N. (2014). First reported case of a squamous cell carcinoma arising in the duodenum in a patient with lynch syndrome. Int. J. Clin. Exp. Pathol..

